# Summation in the Hippocampal CA3-CA1 Network Remains Robustly Linear Following Inhibitory Modulation and Plasticity, but Undergoes Scaling and Offset Transformations

**DOI:** 10.3389/fncom.2012.00071

**Published:** 2012-09-25

**Authors:** Dhanya Parameshwaran, Upinder S. Bhalla

**Affiliations:** ^1^National Centre for Biological Sciences, Tata Institute of Fundamental ResearchBangalore, India

**Keywords:** linear summation, network computation, robustness, input–output transformation

## Abstract

Many theories of neural network function assume linear summation. This is in apparent conflict with several known forms of non-linearity in real neurons. Furthermore, key network properties depend on the summation parameters, which are themselves subject to modulation and plasticity in real neurons. We tested summation responses as measured by spiking activity in small groups of CA1 pyramidal neurons using permutations of inputs delivered on an electrode array. We used calcium dye recordings as a readout of the summed spiking response of cell assemblies in the network. Each group consisted of 2–10 cells, and the calcium signal from each cell correlated with individual action potentials. We find that the responses of these small cell groups sum linearly, despite previously reported dendritic non-linearities and the thresholded responses of individual cells. This linear summation persisted when input strengths were reduced. Blockage of inhibition shifted responses up toward saturation, but did not alter the slope of the linear region of summation. Long-term potentiation of synapses in the slice also preserved the linear fit, with an increase in absolute response. However, in this case the summation gain decreased, suggesting a homeostatic process for preserving overall network excitability. Overall, our results suggest that cell groups in the CA3-CA1 network robustly follow a consistent set of linear summation and gain-control rules, notwithstanding the intrinsic non-linearities of individual neurons. Cell-group responses remain linear, with well-defined transformations following inhibitory modulation and plasticity. Our measures of these transformations provide useful parameters to apply to neural network analyses involving modulation and plasticity.

## Introduction

The characterization of input–output (*I*–*O*) transformations of neurons is a key step in tying together connectivity data with network properties. Despite considerable progress in understanding the biophysics of single neurons (Koch and Segev, 2000; Magee, 2000), their responses in a network context with high activity remain difficult to estimate due to complex summation of multiple excitatory and inhibitory inputs, as well as plasticity.

The hippocampal CA3-CA1 network has a simple feed-forward projection circuit and is believed to play a role in hetero-associative memories (Rolls, 2010). This function relies on weighted linear summation of multiple inputs to hippocampal neurons. However, hippocampal CA1 neurons and other pyramidal neurons are known to integrate sub-threshold inputs in a linear or non-linear manner depending on the spatio-temporal nature of the inputs that the dendrites receive (Cash and Yuste, 1999; Polsky et al., 2004; Gasparini and Magee, 2006; Losonczy and Magee, 2006; Spruston, 2008; Branco et al., 2010).

In contrast, network computation theories consider convergence of hundreds of excitatory and inhibitory synaptic inputs, culminating in the highly non-linear thresholding operation of spiking. Upon spiking all analog information about the inputs embodied in the EPSPs as a result of dendritic integration gets digitized. Does the neuron lose all analog input information as a result of thresholding? Many network theories simply discard neuronal spiking and treat “units” as analog summation entities, which may be linear (McCulloch and Pitts, 1943). Other analyses consider population averages of spiking (Gerstner, 2000; Rasch et al., 2009). In each case a common assumption is the transformation of spiking activity of cells into some analog code. The current study addresses the question: Do real neural networks exhibit such population analog activity, and is this encoding a linear transformation of inputs?

Even within the assumptions of linearity, network properties such as sensitivity to input and ability to propagate depend on the input–output transformation parameters (Salinas and Abbott, 1995; Holt and Koch, 1997; Chance et al., 2002; Rothman et al., 2009). While there is a wealth of data on these modulations at the sub-cellular and single-neuron level (Turrigiano and Nelson, 2000; Chance et al., 2002; Rothman et al., 2009), it is important to establish what happens to spiking properties of cell groups undergoing network-level modulation.

In the current study, we stimulate upstream CA3 axons using an electrode array to give synchronous, near-threshold synaptic inputs at multiple sites on CA1 neurons. We monitor summed, multi-neuron calcium responses, and show that these provide a readout of spiking and exhibit a linear summation of inputs across the recorded CA1 cell groups. We show that this linear summation rule remains robust and obeys consistent scaling rules for different network contexts, including modulation of activity, inhibition, and synaptic plasticity.

## Materials and Methods

All of the experimental procedures were approved by the National Centre for Biological Sciences institutional animal ethics committee, in accordance with the guidelines of the Government of India.

### Dye loading

Four hundred micrometers of transverse hippocampal slices were prepared from 4 to 6-week-old male Wistar rats using a vibratory microtome (Vibratome 1000 classic series, Vibratome, USA) in ice-cold artificial cerebro-spinal fluid (*a*CSF) containing (in mM) – 118 NaCl, 2.5 KCl, 2.5 CaCl_2_, 1.25 MgSO_4_, 1.25 NaH_2_PO_4_, 26 NaHCO_3_, and 10 glucose, saturated with 95% O_2_/5% CO_2_. Slices were equilibrated in *a*CSF at room temperature for 120 min.

Slices were loaded using ballistic delivery of fluorescent dye. Ballistic loading sparsely loaded tens of neurons with the dye in comparison to the AM-ester dyes where many hundreds of neurons get loaded simultaneously. Calcium-green-1 dextrans conjugated dye (Molecular Probes C-6765) was coated on gold particles (1–1.5 μm radius, Aldrich 326585) and delivered into the slice preparation with a “gene-gun.” This method results in loading of individual cells contacted by these particles (Kettunen et al., 2002). Metal filters were used to protect the tissue from shock wave generated by the gun at high pressure (60–80 psi).

**Figure 1:**
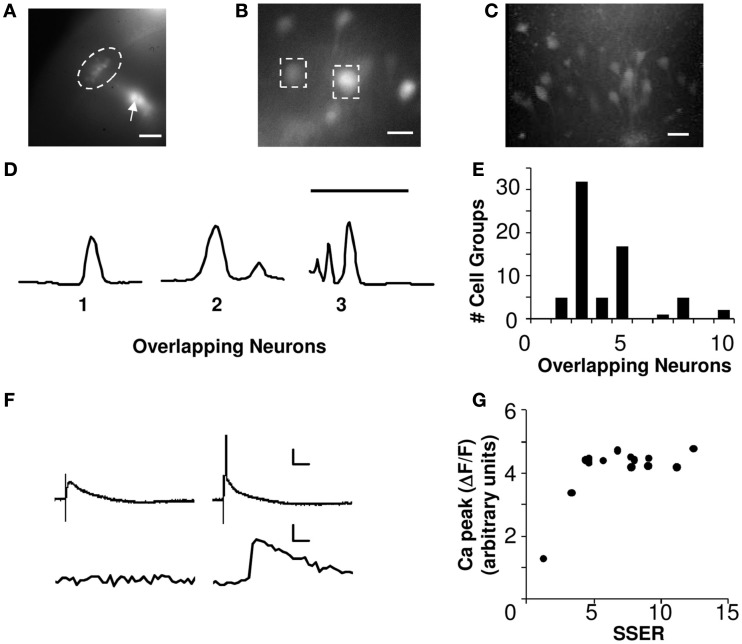
**Calcium signals report spiking of small cell groups**. **(A)** Fluorescence image showing CA1 neurons (encircled by the dotted line) loaded with Calcium-green-1 dextrans (10×, scale bar 100 μm). The arrow points toward a clump of dye-coated gold particles. **(B)** Dye-loaded CA1 somas imaged at 40× (scale bar 20 μm). The dashed boxes around the neurons show two ROIs. **(C)** Z-stack of 2-Photon image of CA1 neurons loaded ballistically showing low overlap (scale bar 50 μm). **(D)** Intensity profile of 2-photon Z-stacks for three ROIs. The number of peaks in the intensity profile is a readout of the number of overlapping neurons (scale bar 100 μm, *Y*-axis represents intensity). **(E)** Histogram of the overlapping neurons in each cell group estimated using 2-photon Z-stack images of ballistically loaded slices, and scaled to the full slice thickness (*N* = 5 slices). On an average each ROI was a readout from 4.1 neurons. **(F)** Show two trials of simultaneous whole-cell patch voltage (above) and calcium (below) recordings from CA1 neurons. Calcium activity correlates with the spiking response. In the voltage trace the initial glitch represents the stimulus artifact. Panel on the left shows a non-spiking EPSP response and no calcium response. Panel on the right shows spiking responses and the corresponding calcium activities (scale bar *X*-axis 5 ms, *Y*-axis 20 mV, 1% Δ*F*/*F*, cell #130710s1c1). **(G)** Calcium peak response from an example neuron to a series of increasing input strengths. *X*-axis represents summed single electrode responses (SSER). Calcium response correlates with neuronal spiking (Both *X*, *Y*-axes represent arbitrary units, cell #220710s1c1).

An Olympus microscope (IX 50) with fluorescence attachment was used to image the labeled structures. Three objectives 10×, 40× (oil immersion objective), and 60× (oil immersion objective) were used in the study to get various levels of spatial resolution (Figures 1A,B). Videos were captured on a high speed cooled CCD camera (Andor DV iXON 887 BI) at 122 Hz.

### Analysis of neuronal overlap

In an independent dataset, we loaded hippocampal slices ballistically as described above. We imaged Z-stacks of the loaded CA1 neurons using a custom-made two-photon microscope (Figure 1C). Two-photon exposure times of ballistically loaded slices were limited because of heating of the gold beads. Therefore the ballistically loaded slices could not be imaged during the actual experiment using a two-photon microscope. The intensity profile along the depth of the Z-stacked images was plotted for 67 regions of interest (ROI; Figure 1D). The number of peaks and the width of the intensity profile (<30 μm was considered as a single neuron) was used as a measure of number of overlapping neurons in each ROI. We obtained Z-stacks for an average depth of 150 μm of healthy tissue. Our full slice thickness was 400 μm, and by excluding the bottom and top 50 μm we estimate it had a total of 300 μm of healthy slice. We therefore scaled our cell counts for each ROI by a factor of ∼2 to estimate the total number of loaded cells in the slice (5 slices, 67 ROIs). The scaling was based on the assumption that the distribution of loaded neurons remained uniform across the cross-section of the slice. Our extrapolation for 67 ROIs yield an estimated range of 2–10 cells per ROI (on an average a readout from 4.1 neurons/ROI) as recorded by our CCD camera (Figure 1E).

### Current calibration and input protocol

The stimulating electrodes consisted of an array of 3–5 twisted bipolar electrodes (Nichrome, 50 μm outer diameter). The electrodes were arranged in a straight line and placed along the dendritic axis (*Y*-axis).

We calibrated all stimulating electrodes using fluorescence responses and field EPSP recordings. We adjusted currents for minimal overlap between axons stimulated by different electrodes by using cross-electrode paired-pulse stimulation (Creager et al., 1980).

These currents were fixed for the rest of the experiment, including the high frequency stimuli (HFS) stimuli used for LTP (19 slices, 217 cell groups). In some experiments we reduced the current to 0.75 of the reference value in order to deliver lower-amplitude stimuli (6 slices, 48 cell groups).

Input patterns were delivered using a Master-8 (A.M.P.I.). Each pattern was a single pulse of current (60 μs) delivered synchronously through several electrodes. The network was stimulated with all possible binary combinations $\left(2^{\text{NEL}}-1\right)$ of the inputs with *N*_EL_ electrodes and the normalized fluorescence responses were recorded. We were able to deliver a maximum of 31 patterns with five electrodes. Each input pattern was repeated for three trials. LTP was induced by using a three HFS (100 Hz for 1 s) pattern with inter tetanic interval (ITI) of 300 s (spaced tetanic stimuli; 9 slices, 65 cell groups). The potentiated network responses were recorded at least 15 min after the last tetanic stimuli in order to give the responses time to stabilize.

### Fluorescence measurements

Regions of interest were selected as rectangular areas around the dye-filled neuron. The size of the ROI was ∼20 μm × 20 μm. All responses were calculated as a mean change in fluorescence amplitude measured as Δ*F*/*F* in the ROI.

(1) $$\frac{\Delta F}{F}=\frac{\left(F_{\text{peak}}-F_{\text{baseline}}\right)}{F_{\text{baseline}}}$$

Where *F*_baseline_ is the mean fluorescence measured over 500 ms baseline before the stimulus is induced, *F*_peak_ is the maximum fluorescence recorded in the 100 ms window after the stimulus. We also computed area under the calcium curve. The area under response curve was calculated in the 100 ms window after the stimulus. As the area was proportional to the peak, we used the simpler peak estimate for all our analyses.

### Linear summation models

The calcium response of a single neuron can be defined as

(2) $$\begin{array}{c} \text{Single}\;\text{neuron}\;\text{response},r_{j}=0,\;\text{if}\;f\left(I∙w\right)<T_{j}\\ =1,\;\text{if}\;f\left(I∙w\right)>T_{j} \end{array}$$

where *r_j_* is a binary variable that represents the calcium response of a single neuron *j*, *T_j_* is the spiking threshold for neuron *j*, and *w* is neuron specific synaptic weights vector of neuron *j* for a given binary input vector *I*. The function *f*(*I*•*w*) is the transform between the dendritic and somatic membrane voltage at each CA1 neuron.

We assumed that each cell group consisted of 2–10 neurons, each of which could respond to stimuli with a single spike if the combined inputs crossed threshold (Eq. 2). The fluorescence change recorded from a cell group is the sum of the fluorescence change of individual neurons.

(3) $$\text{cell group output},\;O=\sum_{j=1}^{\text{Neurons}}{\left(\Delta F/F\right)}_{j}\cdot r_{j}$$

(Δ*F*/*F*)*_j_* represents the normalized fluorescence change associated with neuron *j*, *r_j_* represents whether the neuron spikes and *O* represents the overall fluorescence change recorded in the ROI/cell group.

We used two models to analyze linear summation in the CA1 cell groups to the 31 input patterns delivered through the five electrodes (Figure 3). These models apply to cell groups and do not imply linearity in the constituent cells.

Model 0: weights were determined by optimizing to get the best fit for linear weighted summation:

(4) $$O_{k\text{ estimated}}=\sum_{\text{input electrodes}i}I_{i}\cdot \;W{\left(k\right)}_{i}$$

(5) $$\text{Error}=\sum_{\text{input patterns}}{\left(O_{k\text{ actual}}-O_{k\text{ estimated}}\right)}^{2}$$

Where *I_i_* represents binary input to electrode *i* and *O_kactual_* represents the calcium response of cell group *k*. The weight vector *W*(*k*) specific to cell group *k* was calculated in order to minimize the error in the estimated output.

Model 1: here we compared the observed response to multi-electrode input to the linear sum of responses to single electrode inputs. Since the precise value of the input from a given electrode is a complex composite of electrode geometry, current, axons stimulated, and synaptic weights of these axons onto target neurons, we were not able to measure it directly. Instead we used the efficacy of this electrode in eliciting an output on the target cell group as an indirect but consistent measure of the effective input that incorporated all these factors. Specifically, we defined the optically measured response of a given cell group to the stimulus delivered at a single electrode as the single electrode response (Eq. 3). Using this as a basis, we could express the effective input delivered at multiple electrodes by summing up the contributions of each single electrode. We used the summed single electrode response (SSER)

(6) $$\text{SSER}=\underset{\text{input electrodes }i}{\sum }O_{i}$$

as a surrogate for the total input delivered on multiple electrodes, and for subsequent tests for linearity. *O_i_* represents recorded calcium response to input through a single electrode *I* for a given cell group. SSER was calculated independently for each cell group.

Linear systems are mathematically characterized by two properties – scaling and superposition. If *y* = *f*(*x*) represents a linear system then it should satisfy the following criteria:

1. Scaling, *y*1 = *f* (*x*1) then *a*·*y*1 = *f* (*a*·*x*1) where *a* is a constant.
2. Superposition, *y*1 = *f* (*x*1) and *y*2 = *f* (*x*2) then *f* (*x*1 + *x*2) = *f* (*x*1) + *f* (*x*2).

We first analyzed system linearity by comparing the actual responses (Δ*F*/*F*)*_ij_* to the SSER (from Eq. 6) over all permutations of inputs (for 31 data points delivered through five electrodes). We refer to this as the *I*–*O* transform curve. According to the above mathematical characterization, SSER represents the sum of responses over different combinations of *x* individual inputs $\left(\text{or}\;\underset{i}{\sum }f\left(xi\right)\right),$ and the actual measured response (Δ*F*/*F*) represents $f\left(\underset{i}{\sum }xi\right).$ If the *I*–*O* curve remains along a straight line with a slope of 1, then the summation is perfectly linear. We used Model 0 and Model 1 to test for the linearity. We also analyzed the scaling feature of linearity, by reducing the inputs. Here we used the scaling factor *a* = 0.75.

In addition to the analysis of system linearity, we also employed the linear regression statistic to study slope transformations. Here we use the term linear fit to refer to a tight fit using the linear regression statistic. If a single electrode input did not elicit a response, its response was assigned as zero. All data fitting in the *I*–*O* transform curves was done using a linear regression statistic. The *I*–*O* data was fit to a straight line passing through zero. Data points with zero calcium response and zero SSER were not considered while calculating the regression fit. This gave us the slope measure. The cell group was classified as linear if the scatter was low (*R*^2^ > 0.75). Scatter was calculated as follows

(7) $$R^{2}=\frac{1-{\sum \left[{\left(\Delta F∕F\right)}_{ij}-\text{Regression}\;\text{slope}\;x\left(\sum W_{ij}I_{i}\right)\right]}^{2}}{\sum {\left(\Delta F∕F\right)}_{ij}^{2}}$$

To look for changes in slope, we calculated log(Output/SSER) for each data point normalized according to Model 1. Significance was calculated using Student’s *t*-test. To look for equivalence in slope, we used the Student’s *t*-test as above (*p* > 0.05). Additionally we checked whether the regression slope after network perturbation was within 95% confidence intervals of the regression fit prior to perturbation.

All analysis was done using MatlabR2007.

## Results

We measured calcium responses of multiple CA1 neurons to summed synaptic input. We investigated two attributes of linearity in the summed responses. First, are these summed spiking responses linear? Second, how do these responses scale when network parameters change due to synaptic plasticity and inhibitory modulation?

### Calcium signals report spiking of small cell groups

We positioned an array of five stimulating electrodes on the Schaffer collaterals (SC) of rat hippocampal brain slices. In each slice we recorded from 7 to 20 CA1 pyramidal cell groups, using calcium dye recording. The slices were ballistically loaded with calcium-green-1 dextrans (Kettunen et al., 2002), and imaged using an EMCCD camera (see Materials and Methods; Figures 1A–C). The ballistic loading technique strongly loads relatively few cells, leaving others unaffected. Our readouts were from small cell groups comprising of 2–10 neurons (see Materials and Methods; Figures 1D,E). Each ROI was an average readout from ∼4 neurons.

We used peak calcium response as readout to record single neuron and network integration of synaptic inputs. Calcium responses have been reported to correlate with the action potentials (Smetters et al., 1999; Yaksi and Friedrich, 2006). We used simultaneous calcium dye imaging and whole-cell patch recordings from single CA1 neurons to test this result. In our preparation, somatic calcium responses correlated with spiking response and were not visible at small cellular depolarizations (Figure 1F). Furthermore, the peak calcium response from individually patched neurons was a step-function that did not rise further with increased input strength in our stimulus range (Figure 1G). This set a minimum threshold of cellular activation that could be detected in our measurements.

### Synaptic input elicits distinct calcium responses from small cell groups

**Figure 2:**
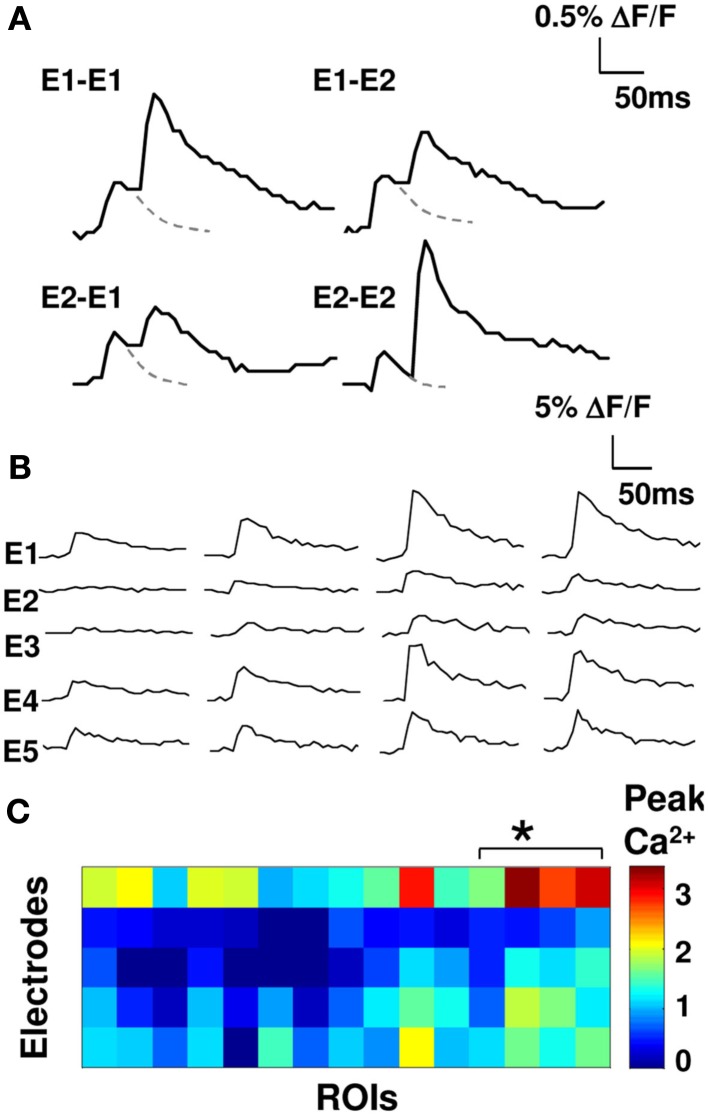
**Synaptic input elicits distinct calcium responses**. **(A)** Overlap between axons stimulated using different electrodes was tested by measuring fluorescence responses to paired-pulse stimuli (50 ms interval). Examples with two electrodes (E1, E2) shown. Dashed line is extrapolated fluorescence decay curve for a single pulse. Cross-electrode facilitation is low (cross-electrode facilitation 1.04 ± 0.04 SEM; same-electrode facilitation of 3.5 ± 0.5 SEM, *p* < 0.05). **(B)** Four representative single trial fluorescence traces in response to stimulation on five electrodes. **(C)** Color-coded matrix of peak calcium responses (five electrodes and 15 cell groups). The asterisk indicates the four neurons from **(B)**.

We stimulated our electrodes one at a time and measured calcium signals to obtain weight matrix (see Materials and Methods; Eqs 1 and 2; Figure 2). This gave us a lumped weight matrix *W*, where each entry represents the effective weight of many synapses converging onto the small group of cells in our calcium readout (see Materials and Methods). We tested for overlap between fiber bundles stimulated using cross-electrode paired-pulse facilitation and found that overlap was small. The cross-electrode facilitation was 1.04 ± 0.04 SEM, whereas the same-electrode facilitation was 3.5 ± 0.5 SEM, *p* < 0.05; see Materials and Methods; Figure 2A; Creager et al., 1980). Our weight matrices were stable over time. We repeated our weight matrix estimation process over a period of 100 min and observed <10% drift. We observed a wide range of effective weights, indicating that the synaptic connections were inhomogenous on the scale of the axon bundles we stimulated, and the small cell groups we monitored (Figures 2B,C).

### Summation: A linear model fits responses to input combinations

**Figure 3:**
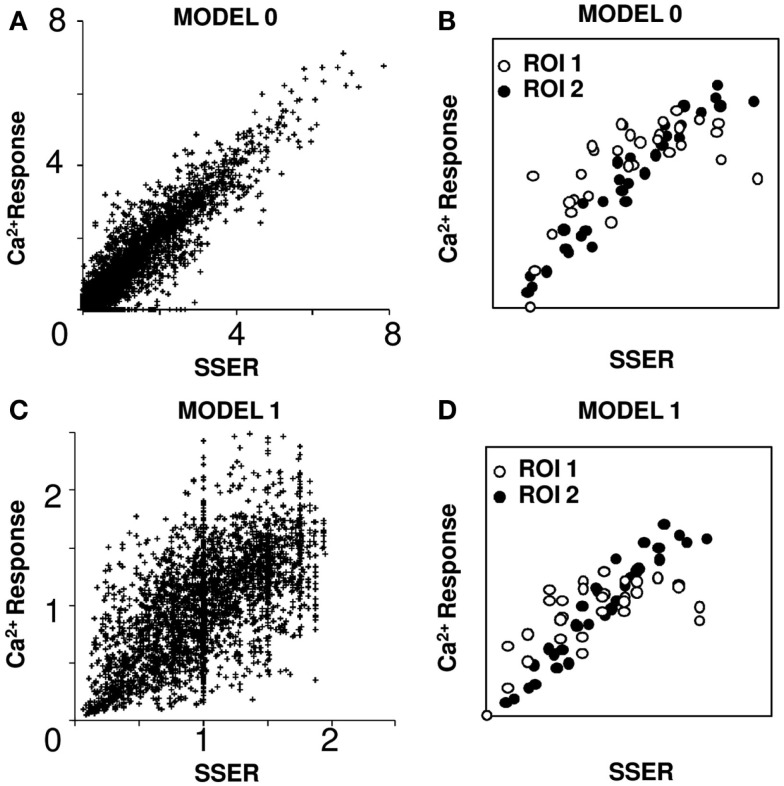
**Linear model fits responses to input combinations**. SSER plotted on the *X*-axis represents summed single electrode response in all plots. **(A)** Model 0: weights were optimized to get a linear fit. Ninety-one percent of ROIs responses can be fit using linear regression. **(B)** *I*–*O* plot for two example ROIs fit using Model 0. **(C)** Model 1: simple linear model in which the calcium responses to all input patterns are normalized to the mean calcium response for each ROI. Seventy-eight percent of single ROI responses can be fit using linear regression. **(D)** *I*–*O* plots for two example ROIs fit using Model 1 [same ROIs as in **(B)**]. Both calcium response and SSER are dimensionless, similar to Δ*F*/*F*.

We next asked if a simple linear summation model could account for responses to all combinations of inputs. As we stress in the discussion, our readouts and inputs were to cell groups, and therefore linearity in these groups does not necessarily imply linearity of summation at the single-neuron level. Using five electrodes we could generate 31 distinct input patterns at a given stimulus amplitude (see Materials and Methods). A simple linear weighted summation rule has the form:

(8) $$O_{j}=\sum I_{i}\cdot W_{ij}$$

where *O_j_* is the estimated output of the *j*th cell group, *I_i_* specifies the *i*th input, and *W_ij_* is the connection weight. There are several ways to estimate connection weight matrix *W* given the 31 input combinations in our dataset. One approach, which weights each of the 31 combinations equally, is to do a least-squares minimization calculation to find the optimal set of weights *W* that will fit all 31 points to a straight line (see Materials and Methods). Using this method we obtained a tight linear regression fit (*R*^2^ > 0.75) for 91% of ROIs around the 45° line (overall fit of all points *R*^2^ = 0.92, slope = 1.01). We thus conclude that the actual response scales with the SSER (see Materials and Methods; Eq. 6) close to the 45° straight line, qualifying the summation as linear (see Materials and Methods). We refer to this as Model 0 (Figures 3A,B).

A less numerically ponderous approach is to read weights directly from the fluorescent responses of a cell group to a given input. Again, in order to balance the contributions of each of the 31 combinations of input, we normalized the outputs to the mean response of all input patterns. This gave a good linear regression fit in 78% of the cell groups (*R*^2^ > 0.75 for a linear regression fit and overall slope of 0.95; see Materials and Methods; Figures 3C,D). Again, the actual response scales linearly with the SSER (see Materials and Methods; Eq. 6). We refer to this as Model 1. We used Model 1 for all further analyses of *I*–*O* transform.

We also investigated several more complex models of summation, including a non-linear conductance-based model (see text; Figure S2 in Supplementary Material). These did not improve on these fits, but were valuable in confirming that the responses of most cell groups were well approximated by a weighted linear sum of inputs for the physiological dynamic range before the response saturates.

### Summation remains linear at reduced levels of excitatory input

**Figure 4:**
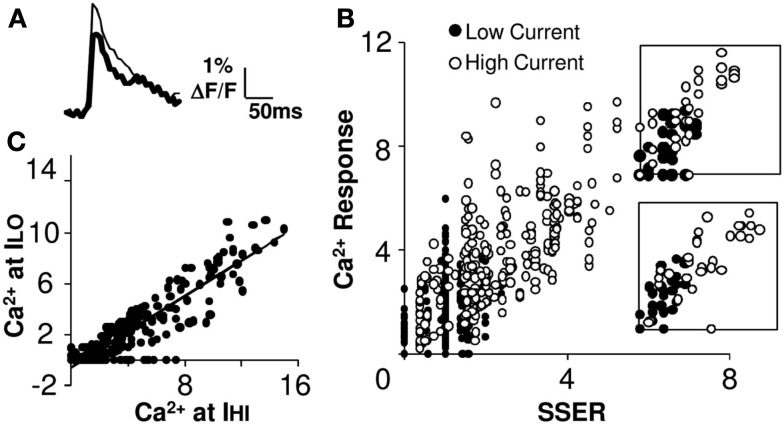
**Summation remains linear at reduced levels of excitatory input**. **(A)** Example fluorescence traces of the response to low (thick) and high (thin) currents. **(B)** Calcium responses from 15 cell groups in slice #100308s2 plotted against SSER (Model 1) at low (filled circles) and high (empty circles) currents (I_LO_ = 0.75 I_HI_) for 31 input patterns. The two distributions appear continuous with each other, suggesting linearity of input summation. Insets for two cell groups are shown. **(C)** Comparison of Ca^2+^ responses at basal (high) and reduced input stimulus currents. The scatter plot is linear with the exception of several points where the reduced stimulus was below threshold. The best fit line has a negative offset on the *Y*-axis. Both calcium response and SSER are dimensionless, similar to Δ*F*/*F*.

We next delivered a second set of input stimuli, where the current on each electrode was scaled down by the same factor (0.75). The Ca^2+^ responses of the reduced stimulus (*I*_LO_ = 0.75 *I*_HI_) patterns fell around a straight line (Figures 4A,B), which was truncated at zero because many of the reduced responses were below threshold. We found that the slope of the *I*–*O* curve did not change significantly in 67% of the cell groups (see Figure 4B; 95% confidence intervals; Materials and Methods). Reduced stimulation current is expected to activate smaller numbers of axons. A comparison of the calcium responses to the same input patterns before and after the reduction of current gave us a straight line with a negative *y*-offset and a slope of 0.7 that closely matched the input current scaling factor of 0.75 (linear regression fit, *R*^2^ = 0.81). This supports the scaling property of linear summation. Thus the same linear summation rule applied when smaller numbers of input synapses were activated on each electrode (Figure 4C).

In summary a reduction of input number maintaining the excitation-inhibition ratio causes a reduction in responses but does not affect the gain of the *I*–*O* relationship. This provides evidence for the scaling property in linear systems. It should however be noted that the scaling property breaks down when the response of the cell group falls below spiking threshold.

### Summation remains linear with the same slope, when inhibition is blocked

**Figure 5:**
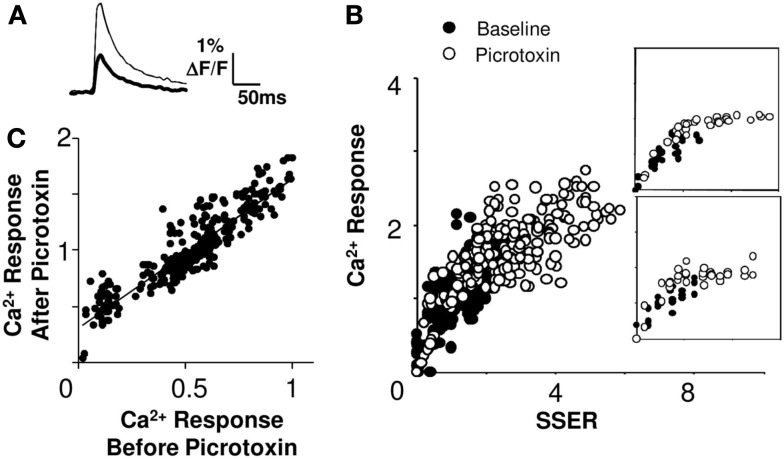
**Summation remains linear at different levels of inhibitory input**. **(A)** Single trial calcium response traces before (thick) and after (thin) application of picrotoxin. **(B)** Calcium responses (Δ*F*/*F*) to the same input before (filled circles) and after (empty circles) the application of picrotoxin. The *I*–*O* curve remains linear for a large portion of the stimulus range, and then saturates (slice #180808s1). Insets for two cell groups are shown. **(C)** Comparison of Ca^2+^ responses before and after picrotoxin application. The relationship is linear (regression fit *R*^2^ > 0.87). The post-picrotoxin response is larger and there is a positive *y*-offset due to spiking of previously inhibited cells. The best fit line has a positive offset on the *Y*-axis. Both calcium response and SSER are dimensionless, similar to Δ*F*/*F*.

The CA1 network includes a substantial number of inhibitory inter-neurons (Megias et al., 2001). We tested the role of inhibition in network responses by applying the GABA-A blocker picrotoxin (20 μM, 7 slices, 40 cell groups) in the bath. This treatment increased neuronal responses (Figure 5A). We analyzed linearity by repeating the combinatorial input patterns in the presence and absence of picrotoxin. It should be noted that on blocking inhibition the network activity tends saturate at higher inputs. We used two approaches to test if the network summation rules changed with inhibition, within the non-saturating range. First, we plotted the pre-picrotoxin and post-picrotoxin responses (inhibition blocked) against SSER estimated using Model 1 (Eq. 8). We found that 78% of cell groups integrated inputs in a linear weighted manner even when inhibition was blocked (*R*^2^ > 0.75 for a linear regression fit; Figure 5B). Furthermore, the slope of the *I*–*O* curve did not change significantly in 78% of these cell groups after the inhibition block (within 95% confidence intervals; see Materials and Methods). Second, we plotted post-picrotoxin responses against pre-picrotoxin responses. Eighty-one percent of these response curves were linear (*R*^2^ > 0.75 for a linear regression fit, Figure 5C). This analysis does not depend on any of our input–output models. We obtained a positive *y*-offset with this model-independent readout of linearity. The mean offset in calcium response on application of 20 μM GABA-A blocker picrotoxin was 38 ± 33% (mean ± SD) of the maximum baseline response.

We therefore conclude that inhibition does not affect the gain of the *I*–*O* relationship, but instead introduces an offset in responses. We consider the implications of this observation in the discussion.

### Summation maintains a linear fit following plasticity, but undergoes gain control

**Figure 6:**
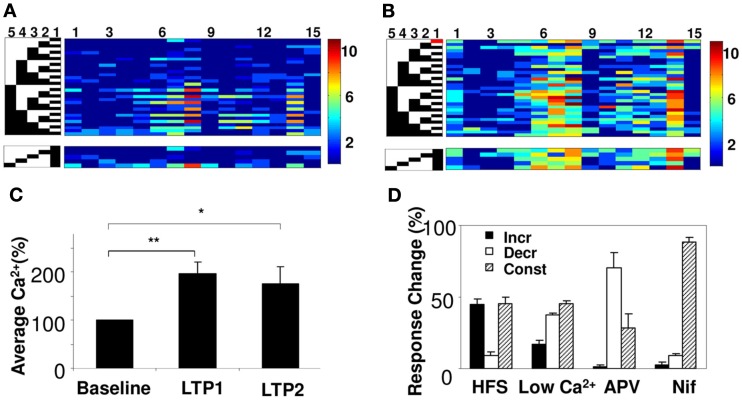
**Network plasticity and summation**. **(A)** Peak calcium response of 15 cell groups to 31 input patterns (left) from five electrodes. Active electrodes are in black. A subset of responses is reordered below to show interactions between electrode 1 and other electrodes. **(B)** Responses of same network following HFS on electrode 1 (red block). Responses increase, but summation properties also change. For example, responses of cell groups 7 and 14 were dominated by electrode 1 before HFS, but become more uniform after HFS. **(C)** Averaged calcium responses increase following repeated HFS (*N* = 13 slices). *p* < 0.005 for LTP1, and *p* < 0.025 for LTP2 using a two-tailed *t*-test. **(D)** Effect of pharmacological agents on single electrode calcium responses upon inducing plasticity. Plot shows the % of cell groups categorized as cell groups whose response increases, decreases, or remains constant. Potentiation decreases on application of 50 μm APV (NMDA receptor blocker), reduced ratio of Ca^2+^ in the extracellular solution to 25% and 10 μm Nifidipine (VGCC-L blocker).

We next examined how synaptic plasticity might affect network summation rules. We did so by modifying synaptic weights using long-term potentiation (LTP) on the SC inputs (Bliss and Lomo, 1973). We first established the baseline response matrix using single-pulse stimuli on each of the five electrodes. We repeated the baseline measurement at least two times. We then induced LTP on one of the electrodes using a spaced HFS protocol with three tetani for 1 s at 100 Hz, separated by 5 min (Ajay and Bhalla, 2004). We waited 15 min for the synaptic weights to stabilize, and then repeated our baseline single-pulse stimuli on each electrode, to record the modified response matrix (Figures 6A,B). In a few cases we induced a second round of LTP on the same electrode. We found that the average calcium responses did not increase further on the second HFS stimulus, similar to the known phenomenon of saturation of LTP (Figure 6C). The properties of plasticity measured with our protocol conformed to known attributes of electrically measured LTP. On blocking NMDA channels using APV and by reducing the ratio of extracellular Ca^2+^/Mg^2+^, the effect of HFS in inducing LTP decreased (Figure 6D).

**Figure 7:**
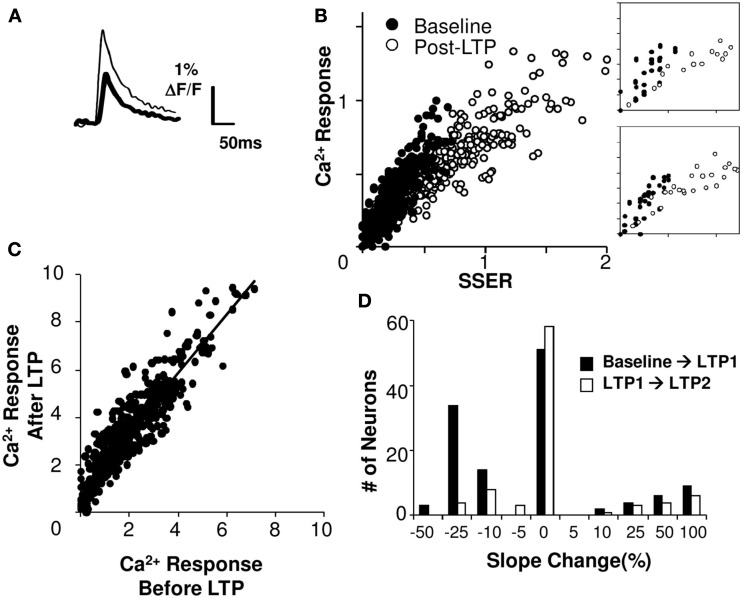
**Summation gain following plasticity**. **(A)** Single trial calcium response traces before (thick) and after (thin) induction of LTP. **(B)** Linear model fit before and after LTP (slice #030108s2). Axes are normalized to pre-LTP input and output ranges. The post-LTP slope (right frame, linear regression fit) is smaller than baseline. **(C)** Model-free comparison of the responses following HFS protocol with responses prior to the stimulation. The best fit line has a positive offset on the *Y*-axis. **(D)** Changes in the slope of the *I*–*O* curve following LTP1 shows a median decrease of 25% (*N* = 113 cell groups). Changes after LTP2 are low. Both calcium response and SSER are dimensionless, similar to Δ*F*/*F*.

Does plasticity change arithmetic rules? We considered two possibilities: that the rule might maintain a linear fit but that plasticity might alter the scaling of neuronal responses to the same inputs, or that the form of the rule itself might cease to be linear. We found that 81% of cell groups integrated inputs in a linear weighted manner even after LTP was induced (*R*^2^ > 0.75 for a linear regression fit). We then carried out an analysis of the slope change in the calcium *I*–*O* curve following LTP (Figures 7A,B). We found that in 43% of the cell groups the slope decreased significantly following LTP, whereas in 13% of the cell groups it increased significantly (*p* < 0.05, using Student’s *t*-test; see Figure 7D; Materials and Methods). The median decrease in the *I*–*O* slope was ∼25%. On further potentiation the changes were much smaller. When we compared calcium responses of individual cell groups before and after LTP, we found that they lay on a tight straight line, with a slope greater than 1 (Figure 7C, linear regression fit, *R*^2^ = 0.83).

Do the input–output parameters change in a manner dependent on the extent of plasticity? Given that the shape of the response remained mostly linear, we estimated the slope and offset for individual cell groups before and after LTP induction. We did not find a strong dependence of slope on the amount of plasticity (Pearson’s correlation coefficient *R* = 0.35, *p* = 0.014). However, the offset in calcium response after plasticity scaled proportionately to the percentage potentiation (Pearson’s correlation coefficient *R* = 0.6, *p* < 10^−5^).

Thus the input–output function remained linear after induction of plasticity, but the gain and offset of the *I*–*O* curve changed.

## Discussion

We have analyzed summation properties of groups of hippocampal CA1 neurons in a network context. To do this, we have characterized peak calcium responses for multiple synchronous input patterns, tested input summation, and examined output transformations. We find that for most such cell groups, a surprisingly simple linear description accounts for the summation of multiple inputs, under many conditions. We have characterized the transformations of the linear input–output functions when we manipulate the number of synaptic inputs, modulate inhibition, and induce plasticity.

### Network vs. single-neuron linearity

Our results demonstrate linear summation of inputs at the level of small cell groups. This contrasts with the extensive literature on non-linear summation within individual neurons, especially for spatially and temporally clustered dendritic inputs (Cash and Yuste, 1999; Koch and Segev, 2000; Gasparini and Magee, 2006; Branco et al., 2010; Lovett-Barron et al., 2012). What does this dichotomy imply for network computation? Given a group of cells with distinct thresholds, distinct synaptic weights, and individual non-linear summation, the combined output signal to a common set of inputs can approximate a linear sum, with a key requirement being that the input weights are not tightly correlated. It is theoretically possible to construct cell groups and summation rules where this does not work (Jolivet et al., 2006; Ostojic and Brunel, 2011). For example, non-linearity emerges from a network where all neurons obey the same non-linear summation rule, if the cells in the group have correlated synaptic weights for their inputs (data not shown). It is therefore significant that, at least in the hippocampal CA3-CA1 neural network, the linear outcome applies. Interestingly, the main selection bias in our cell groups was their spatial proximity. Thus one does not need to invoke specifically interconnected “cell assemblies” to achieve linearity of cell-group responses. Our results show that the inherent heterogeneity among neurons could be sufficient to produce a linear readout of inputs (Koulakov et al., 2002).

This linear result has a useful interpretation for neural network computation. The majority of theoretical results for neural networks assume linear summation (Koulakov et al., 2002; Truccolo et al., 2005; Ostojic and Brunel, 2011). Thus our result suggests that one can apply many theoretical results for neural networks to real networks, with the simple proviso that the neural network results apply to small groups of cells and their lumped synaptic inputs. At the same time, the subtleties of individual neuronal computation may provide another layer of computational capabilities to the real system. For example, one can envision the CA3-CA1 network exhibiting hetero-associative network properties at the cell-group level, while individual neurons obey non-linear dendritic summation and activity-dependent scaling of dendritic excitability (Polsky et al., 2004; Gasparini and Magee, 2006; Spruston, 2008).

In our recordings, we have focused our analyses on the linear summation properties that were found in 78% of the cell groups. However, 22% of the cell groups did not have a good linear fit. This suggests the existence of non-linearities in the CA3-CA1 network. Such non-linearities may be caused due to two reasons – one, these cell groups may have contained small number of cells (1–3 neurons). Two, as suggested by the theoretical model presented above these cell groups may have received correlated synaptic inputs.

### Characterizing transformations of summation rules

Real neurons undergo dynamic modulation of many summation properties, which have been extensively characterized. These extend from synaptic plasticity rules, to local dendritic excitability, activity homeostasis, through to cell-wide neuromodulation (Cash and Yuste, 1999; Turrigiano and Nelson, 2000; Spruston, 2008; Carandini and Heeger, 2011). Such studies typically stimulate individual cells and do not address what happens around them. Some studies have gone further and considered the question of how network-level context affects properties of single cells embedded in the network (Chance et al., 2002; Anastassiou et al., 2011). In the current study we step further back still. Through our readout of spiking activity in small cell groups, we ask how network-level modulation and plasticity affect the distribution of summation properties across local cell groups. While the first-order finding of robust linearity is useful, as discussed above, the specific parameters of input–output transformations are crucial for analyzing network function (Arieli et al., 1996; Koch, 1999; Fernandez and White, 2010; Kumar et al., 2010; Carandini and Heeger, 2011). We have characterized these for three cases.

**Figure 8:**
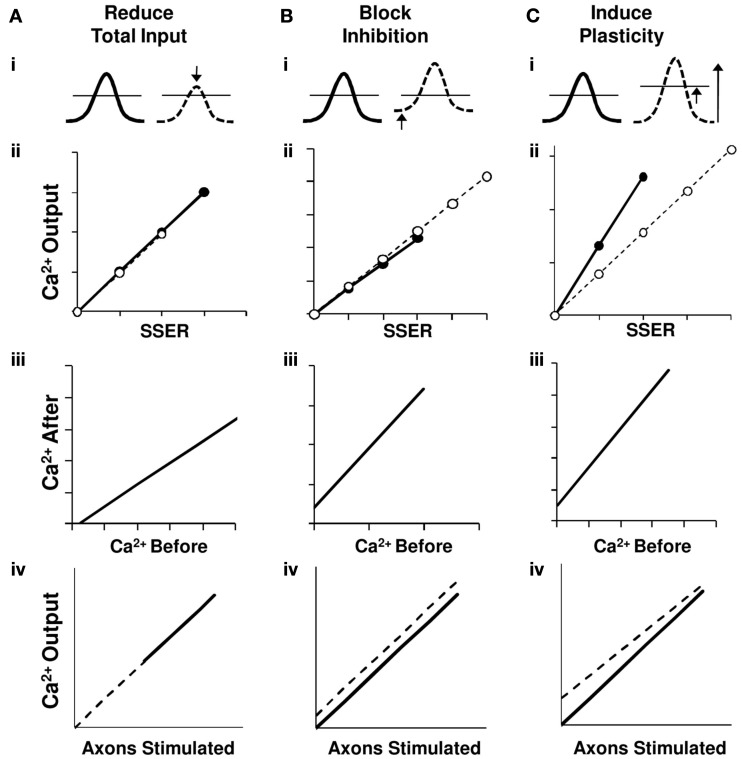
**Network summation hypothesis**. Schematic interpretation of results for the three network perturbations in terms of a linear summation model. Traces on row **(i)** represent summed EPSPs relative to the spiking threshold (horizontal line). Row **(ii)** shows a summary of the calcium responses as a function of normalized total synaptic input (using Model 1) from the perturbation experiments shown in Figures 4B, 5B, and 7B. Row **(iii)** presents the relationship between calcium responses after and before the network perturbations from the perturbation experiment results in Figures 4C, 5C, and 7C. The bottom row **(iv)** represents a schematic of the derived *I*–*O* curves. Here *X*-axis is a correlate of the number of input axons stimulated. In all panels thick lines/filled circles represent response prior to perturbation and dashed lines/open circles represent responses post perturbation. **(A)** On reducing the SSER, the EPSP amplitude decreases causing a downward shift in the *I*–*O* curve without a change in slope. Our data is consistent with additive scaling in this case. **(B)** On blocking inhibition the basal membrane voltage is pushed closer to the threshold, but the size of the EPSP and hence the slope of the input/output line does not change. This too represents an additive shift in the *I*–*O* curve. **(C)** On inducing LTP the input strength increases, resulting in a larger EPSP for the same stimulus. We also see a homeostatic downward multiplicative shift in the *I*–*O* relationship. This can be accounted by an increase in spiking threshold. In all three cases, the input–output relationship remains linear.

First, we show that a balanced reduction of input, maintaining the excitation-inhibition ratio, introduces additive effects on the *I*–*O* relationship. We did not find slope transformation in the *I*–*O* curves in 67% of the cell groups (Figure 8A).

Second, even an unbalanced change that we introduced by blocking inhibitory GABAergic channels also introduces an additive effect in the *I*–*O* curves of 78% cell groups. Our results in spiking CA1 neurons tie in with earlier studies have shown that in the absence of variable background input, inhibition does not cause a gain change (Chance et al., 2002; Fernandez and White, 2010). Additionally, modeling studies have shown that blocking inhibition has an additive effect on spiking cells especially in the case when the inhibitory synapses are proximal to the soma. Mechanistically, the proposed mechanism is that the inhibitory current is limited by the membrane voltage at threshold and can be replaced by a constant offset current in spiking neurons. Thus, unless the excitatory conductance is small compared to inhibitory conductance, inhibition has an additive effect on the *I*–*O* relationship in spiking CA1 neurons (Holt and Koch, 1997). Most of these studies focus their attention on the firing rate changes to asynchronous inputs. Our study confirms that a similar behavior adopted for spiking activity to synchronous inputs in the CA3-CA1 network (Figure 8B).

Third, synaptic plasticity leads to changes both in slope and in offset of the *I*–*O* curve. We find that neuronal summation itself rescales following gain changes in the *I*–*O* relationship that follow plasticity. Changes in gain represent multiplicative effects, as the output response of the cell groups is reduced (or increased) by a factor (Isaacson and Scanziani, 2011). We interpret this as a decrease in intrinsic neuronal excitability rather than synaptic rescaling because our weights are typically larger after the LTP induction (Burrone and Murthy, 2003; Wang et al., 2003; Campanac et al., 2008). This decrease in neuronal excitability may be caused by mechanisms such as increase in spiking threshold or a decrease in the probability of release (Figure 8C). As an extension of this hypothesis, we predict to find a positive gain change with a negative offset in the *I*–*O* transform when LTD is induced. Such homeostatic mechanisms have been proposed to promote network stability (Bear, 1995; Turrigiano and Nelson, 2000).

## Author Contributions

Upinder S. Bhalla and Dhanya Parameshwaran designed the project. Dhanya Parameshwaran did the experiments. Dhanya Parameshwaran and Upinder S. Bhalla analyzed the data and wrote the paper.

## Conflict of Interest Statement

The authors declare that the research was conducted in the absence of any commercial or financial relationships that could be construed as a potential conflict of interest.

## Supplementary Material

The Supplementary Material for this article can be found online at http://www.frontiersin.org/Computational_Neuroscience/10.3389/fncom.2012.00071/abstract
